# A dataset of micro biodiversity in benthic sediment at a global scale

**DOI:** 10.1038/s41597-023-02292-y

**Published:** 2023-06-15

**Authors:** Xumei Sun, Danni Jiang, Yina Shao, Siyuan Zhang

**Affiliations:** School of Marine Sciences, Ningbo University, Ningbo, 315211 People’s Republic of China

**Keywords:** Environmental microbiology, Marine biology

## Abstract

Microorganisms, occupying the largest biomass in deep sea, play essential roles in deep-sea ecosystem. It is believed that the microbes in deep-sea sediments are more representative of deep-sea microbial communities, the microbial composition of which is seldom affected by ocean currents. However, the community of benthic microbes on a global scale has not been adequately explored. Herein, we build a comprehensive global dataset determined by 16S rRNA gene sequencing to characterize the biodiversity of microorganisms in benthic sediment. The dataset comprised 212 records from 106 sites, included sequencing of bacteria and archaea for each site and yielded 4,766,502 and 1,562,989 reads, respectively. Through annotation, a total of 110,073 and 15,795 OTUs of bacteria and archaea were obtained, and 61 bacterial phyla and 15 archaeal phyla were identified, of which the dominant phyla were *Proteobacteria* and *Thaumarchaeota* in deep-sea sediment. Therefore, our findings provided a biodiversity data of microbial communities in deep-sea sediment at global-scale and laid a foundation to further reveal the structures of microorganism communities in deep sea.

## Background & Summary

The largest habitable environments on the earth are located in the ocean, which covers approximately 70% of the earth’s surface and is the largest habitat on the planet^1^. Based on the depth of the ocean below the sea level, the global ocean is vertically divided into five zones, including epipelagic zone (depth <200 m), mesopelagic zone (depth between 200 m and 1,000 m), bathypelagic zone (depth between 1,000 m and 4,000 m), abyssopelagic zone (depth between 4,000 m and 6,000 m) and hadal zone (depth >6,000 m)^2–4^. Generally deep sea is considered to be the area below the epipelagic zone^1^, which possesses the largest aqueous habitat for microbial lives^5,6^. Low temperature, high pressure, and low concentrations of bioavailability nutrient dominate habitable aphotic deep-sea environments^3,7,8^. Although such conditions are often referred to as “extreme”, these environments are actually quite common on a global scale^1^. In deep-sea environments, microorganisms account for the vast majority of marine biomass^9^. Although there are some findings about the composition of global ocean microbes, these reports focus on the microbial communities of epipelagic, mesopelagic and abyssal sea waters^2,10–12^. The microbial biodiversity of global deep-sea sediments has not been extensively explored.

The seawater in the ocean is always in a state of flow with the oceanic currents, whether near the bottom or at the surface^13^. The composition of microbial communities of deep-sea water is usually changed due to the influence of ocean currents. As reported, the microbial communities in seawaters are passively flowed related to the trend of ocean currents and locally structured by environmental conditions^14^. Because of the coupling between surface and bottom, the composition and distribution of bacterial communities near the bottom of the seawater correlate with the flowing surface water^15^. Even in the seawater near the seafloor, the microbial communities are inconsistent with those in the sediments^16^. The analysis of environmental factors reveals that the microbial communities of the sediments, but not the seawaters, can be more representative of the characteristics of the deep-sea location^16^. The microbial composition of seawater cannot represent the typical microbial characteristics of an oceanic site^13,16^. In this context, the microbial composition of deep-sea sediment represents the typical microbial biogeography of the ocean^17^. In deep-sea sediments, there inhabit the majority of our planet^18^. Although some studies have been conducted on the biogeography of deep-sea sediments, the samples used in these studies are few and narrow in scope^19^. The investigation with adequate deep-sea samples is necessary, which can provide deep insights into the lives in the deep-sea sedimentary biosphere.

To explore the biodiversity characteristics of the deep-sea microbial communities and build a comprehensive global dataset, the microorganisms were extracted separately from each of the 106 sediment samples and subjected to bacterial and archaeal 16S rRNA gene sequencing, respectively. Based on the sequencing, annotation and analyses, the dataset of bacterial and archaeal diversity in deep-sea sediment at global scale were conducted, which is great advantage for the investigation of microbial diversity, microbial process, and biogeography in benthic sediments.

## Methods

### Sample collection

**Fig. 1:**
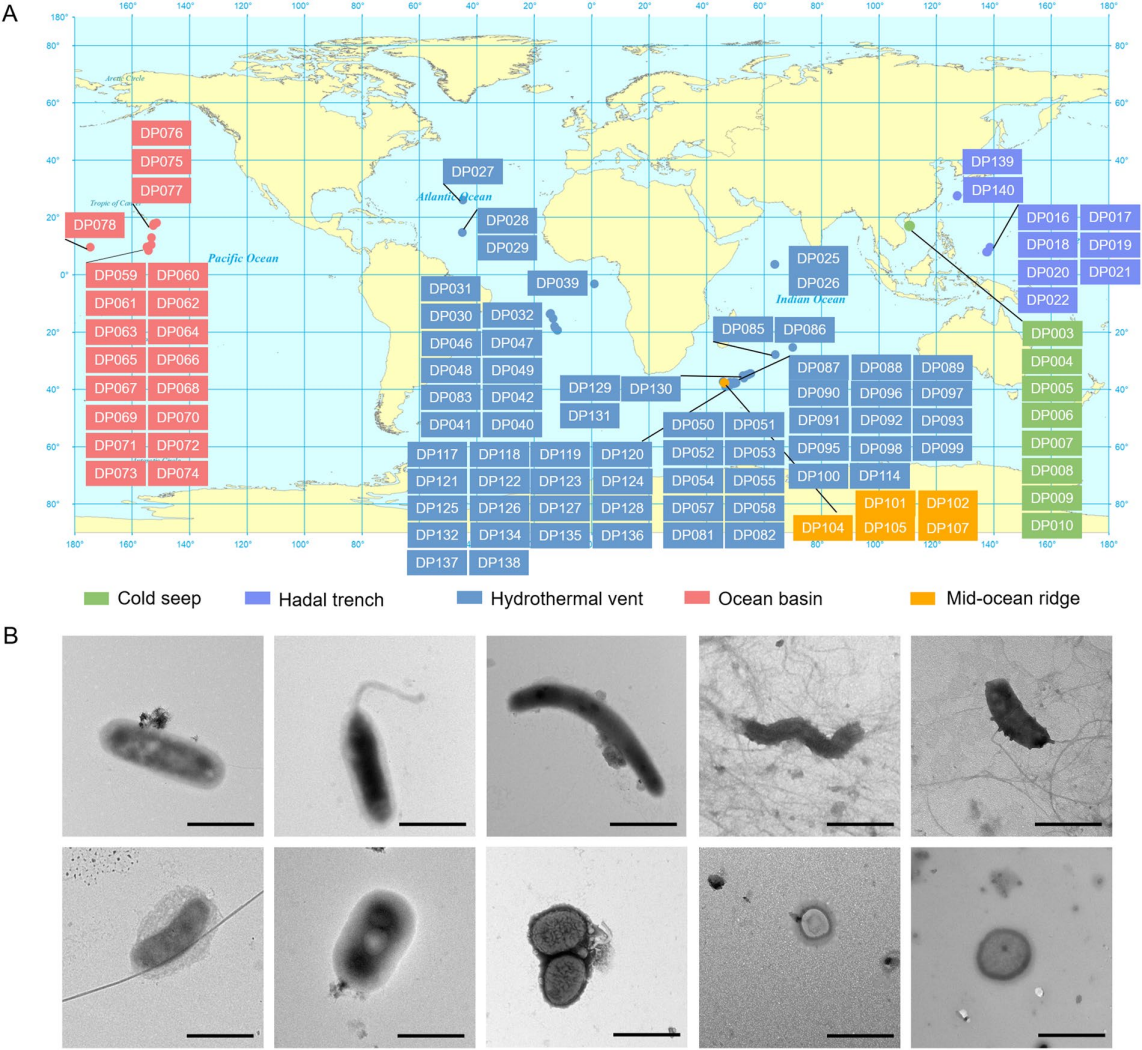
Classification of deep-sea sediments. (**A**) Global distribution of deep-sea sediment samples. Different type of deep-sea environment was represented by different colour. (**B**) Observation of microbes isolated from deep-sea sediments by transmission electron microscopy. Scale bar, 1 µm.

Deep-sea sediment samples were collected from the Atlantic Ocean, the Pacific Ocean and the Indian Ocean using sealable sampling boxes (Oktopus, Germany) during the 22nd, 26th, 30th, 34th, 38th, 39th, 40th and 45th cruises of Oceanic Vessel No. 1. The longitude, latitude, depth and other essential information of each sample is collected along with the voyage, siting of each sample was labelled on the world map in Fig. 1A and the detail information was showed in Table S1 (Fig. 1A and Table S1). The samples contained 5 different deep-sea ecological environments, including hydrothermal vent, cold seep, hadal trench, mid-ocean ridge and ocean basin. The samples were stored at −196 °C until use to prevent the alteration of the original biological community in the sample.

### Isolation of microbes and microscopy

The surface of sample was removed using autoclave sterilized sterile shovel to exclude the exogenous contamination. Deep-sea sediment (20 g) was added with 10 mL sterilized SM buffer (25 mM Tris-HCl, 200 mM NaCl, 20 mM MgCl_2_, pH7.5). After shake for 25 min at 4 °C, the mixture was centrifuged at 100 × g for 5 min and the supernatant was collected. This procedure was repeated 5 times. The last supernatant was centrifuged at 5000 × g for 30 min. The pellet was resuspended with SM buffer as the purified deep-sea microbes. The isolated microbes were observed by transmission electron microscope with negative staining of 2% phosphotungstic acid (pH7.0)^20^. The microbial cells from deep-sea sediments with various morphology, contained bacillus (approximately 2–4 µm in length), spirilla (approximately 200–300 nm in width), vibrio (approximately 1.2 µm in length), and small cocci (approximately 500 nm in diameter) (Fig. 1B), indicating the high diversity of microbiota in deep-sea sediment.

### Extraction of DNA and sequencing

Microbial genomic DNA of deep-sea sediment was extracted using the FastDNA® SPIN kit for soil (MP Biomedicals, USA) following the manufacturer’s protocols. Sequencing was performed using universal bacterial primers 515 F (5′-GTGCCAGCMGCCGCGG-3′) and 907 R (5′-CCGTCAATTCMTTTRAGTTT-3′) covering the V4-V5 regions of bacterial 16S rRNA gene or universal archaeal primers 344 F (5′-ACGGGGYGCAGCAGGCGCGA-3′) and 915 R (5′-GTGCTCCCCCGCCAATTCCT-3′) covering the V3-V5 regions of archaeal 16S rRNA gene^20^. The PCR products were used to prepare the barcoded library using the Illumina Truseq DNA library kit (Illumina, USA). Sequencing was performed using Illumina PE250 (Illumina, USA) and the paired-end reads were overlapped to assemble the V4-V5 tag sequences of bacteria or the V3-V5 tag sequences of archaea using the Flash program.

### Raw data quality control

In order to obtain more accurate results, the raw data was firstly filtered by Trimmomatic v0.33. Then the primer sequences were identified and removed by cutadapt 1.9.1 and a custom Perl script (Supplementary Methods), which finally generated high-quality reads without primer sequences. Based on overlapping sequences, high-quality reads were assembled by FLASH v1.2.7, which generated clean reads. Finally, the chimeric sequences were identified and removed by UCHIME v4.2, and generating effective reads. The sequencing of bacterial and archaeal 16S rRNA gene of 106 deep-sea sediments yielded 4,766,502, and 1,562,989 reads, respectively (Table S2).

### OTU clustering

**Fig. 2:**
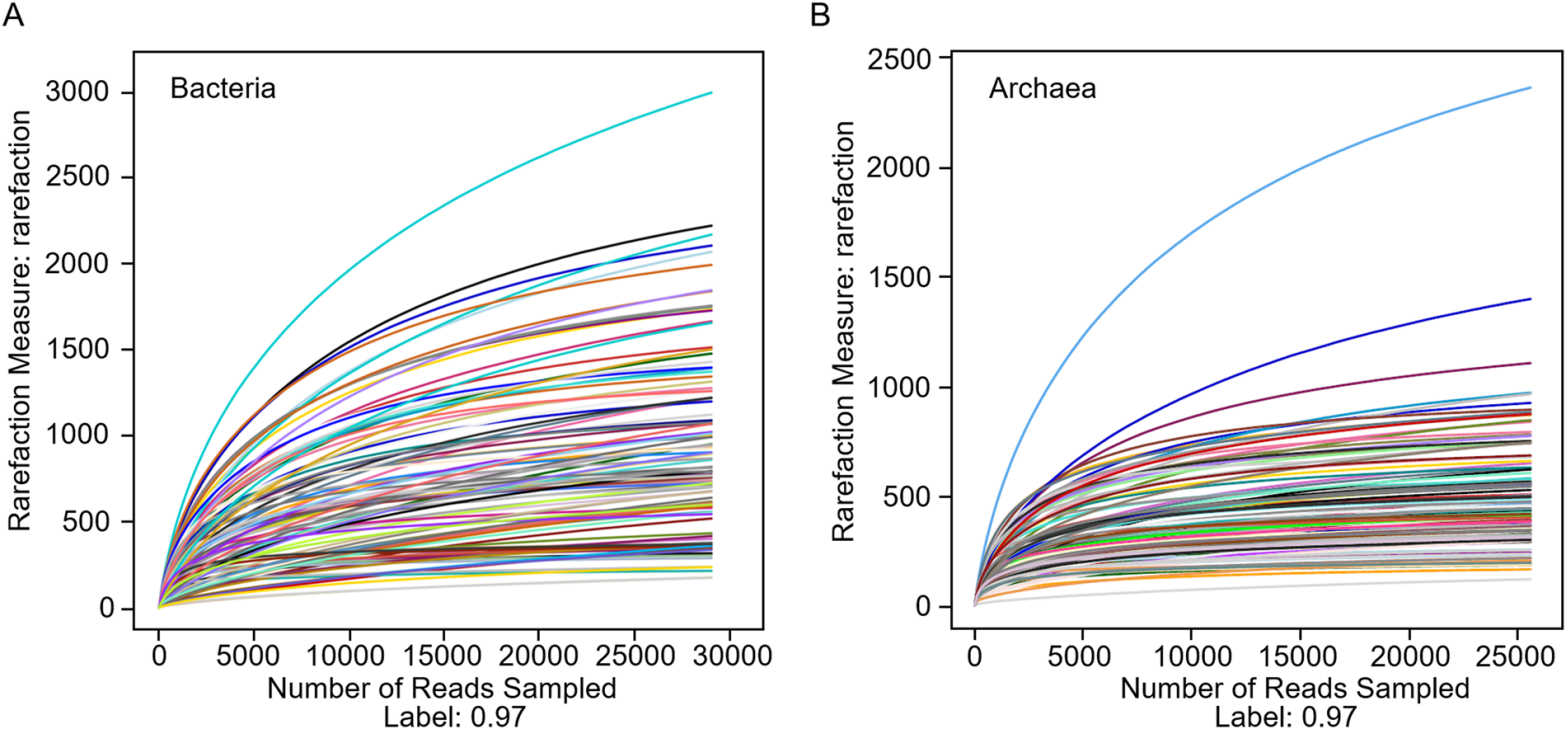
Validity of 16S rRNA gene sequencing data. The plateau rarefaction curves of (**A**) bacteria and (**B**) archaea from 106 deep-sea sediment samples.

**Fig. 3:**
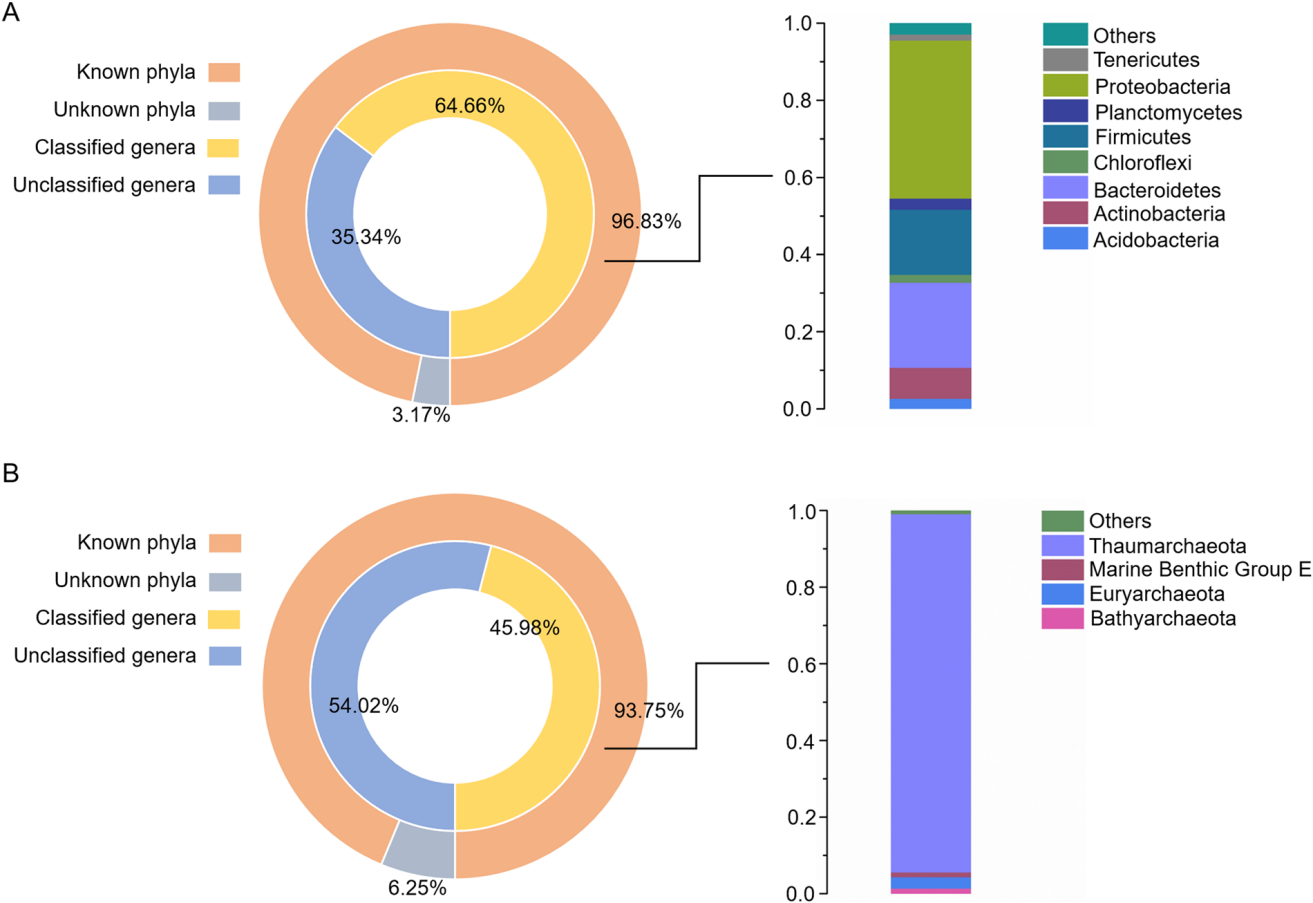
The percentage of classified/unclassified OTUs. (**A**) Pie diagram of the known/ unknown bacterial phyla and classified/unclassified bacterial genera in the deep-sea sediment samples. “Others” represented the phyla with abundance less than 1%. (**B**) Percentage of the known/unknown archaeal phyla and classified/unclassified archaeal genera. The relative abundance of the archaeal phyla was showed on the right. “Others” represented the archaeal phyla with abundance less than 1%.

The candidate sequences were classified into operational taxonomic units (OTUs) by 97% sequence similarity using the Usearch program. Operational Taxonomic Units (OTUs) refers to a cluster of sequences used to define a group (e.g species, genus, strain, etc) in phylogenetic studies or population genetic studies. Sequences with more than 97% similarity were assigned to the same OTUs using USEARCH (version 10 http://drive5.com/uparse/)^21^. A representative sequence for each OTU was annotated with threshold 0.8 using UCLUST v1.2.22q by searching the SILVA database^22,23^. For comparisons between samples, the OTU abundances were normalized by the number obtained from the sample with the lowest counts. The Shannon and Simpson diversity indices and rarefaction curves were generated using the Mothur program^24^. Based on the clustering of OTUs, a total of 110,073, and 15,795 operational taxonomic units (OTUs) were identified (Table S2). The plateau rarefaction curves of 106 samples (Fig. 2A,B) and around 98% coverage of sequencing data were (Table S2) indicated the sequencing data represented the microbial communities of the 106 samples. In total, 61 of 63 (96.83%) phyla matched the known bacteria, and 15 of 16 (93.75%) phyla were matched the known archaea, of which the dominant phyla were *Proteobacteria* and *Thaumarchaeota* in deep-sea sediment (Fig. 3A,B, Tables S3, S4). At the genus level, 1,105 bacterial genera were identified, accounting for 64.66% of the total, and 40 of 87 (45.98%) archaeal phyla were known and could be classified (Fig. 3A,B, Tables S3, S4).

## Data Records

Data obtained from next generation sequencing were uploaded and released on NCBI (national center for biotechnology information) database (Bioproject ID: PRJNA725024, PRJNA725590)^25,26^. Other basic data obtained by sequencing of each sample were shown in Table S2. The morphology of microorganisms under observation by transmission electron microscope were shown in Fig. 1B.

Working links for NCBI records:

https://identifiers.org/ncbi/insdc.sra:SRP316348

https://identifiers.org/ncbi/insdc.sra:SRP318042

## Technical Validation

Microbial genomic DNA was extracted and subjected to 16S rRNA gene sequencing for bacteria and archaea using Illumina PE250 (Illumina, USA) after extracting total DNA of each sediment sample. Sequencing and initial data processing was performed by Mingke Biotechnology Co., Ltd (Hangzhou, China) using universal methods. For details, see Methods.

This dataset contains 106 bacterial and archaeal 16S rRNA gene sequencing records of the 106 deep-sea sediments. All recorded data were collected by Mingke Biotechnology Co., Ltd (Hangzhou, China) and were cross-checked by coauthors, and all uncertainties and discrepancies were discussed by consensus with a third reviewer. The institution that provided the samples re-checked the longitude, latitude, depth and other essential information information to verify the geographic location of each deep-sea sediment. The verification process refers to the same standard as that used in the data entry process. ArcGIS software was used to determine the coordinates of the central points of each sample.

## Supplementary information

Supplementary Information

## Data Availability

No custom code was made during the collection and validation of this dataset.
