# Supplementary material for: A dataset of micro biodiversity in benthic sediment at a global scale
Source: Sci Data. 2023 Jun 15;10:383. doi: 10.1038/s41597-023-02292-y (PMC10272205; doi:10.1038/s41597-023-02292-y)
Supplement: Supplementary file 1 — Supplementary Information [file 41597_2023_2292_MOESM1_ESM.pdf]

Supplementary Information

1. Supplementary Methods
2. Table S1. Sample information.
3. Table S2. 16S rRNA gene sequencing data of bacteria and archaea.
4. Table S3. Classified bacterial genera of 106 deep-sea sediment samples.
5. Table S4. Classified archaeal genera of 106 deep-sea sediment samples.

Supplementary Methods
Custom Perl script

```
#!/usr/bin/perl -w
use strict;

my ($inputFq,$config,$linkPrimer_th,$outPrefix);
#my $reversePrimer_th;

my $opt;
while($opt = shift){
	if($opt eq "-i"){
		$inputFq = shift;
	}elsif($opt eq "-f"){
		$config = shift;
	}elsif($opt eq "-l"){
		$linkPrimer_th = shift;
#	}elsif($opt eq "-r"){
#		$reversePrimer_th = shift;
	}elsif($opt eq "-o"){
		$outPrefix = shift;
	}elsif($opt eq "-h"){
		&usage();
		exit;
	}
}

unless($inputFq and $config and $outPrefix){
	&usage();
	exit;
}

#$barcode_th = 0 unless(defined($barcode_th));
$linkPrimer_th = 2 unless(defined($linkPrimer_th));
#$reversePrimer_th = -1 unless($linkPrimer_th);

my %abbrev=(
	 'A' => "AA",	 'T' => "TT",
	 'C' => "CC",	 'G' => "GG",
	 'M' => "AC",	 'R' => "AG",
	 'W' => "AT",	 'S' => "CG",
	 'Y' => "CT",	 'K' => "GT",
	 'V' => "ACG",	 'H' => "ACT",
	 'D' => "AGT",	 'B' => "CGT",
	 'X' => "ACGT",	 'N' => "ACGT",
	'I' => "ACGT",
);
```

```
my (%length,%sample,%linkPrimer,%Rbarcode);
my %reversePrimer;

open INC,$config or die "can\'t open sample config: $config\n";
while(<INC>){
	next if(/^\#/);
	my @temp = split;
	die "ERROR: F barcode $temp[1] used for more samples\n" if(exists
$sample{$temp[1]}{$temp[2]} and $sample{$temp[1]}{$temp[2]} ne $temp[0]);
	die "ERROR: F barcode $temp[1] has different R barcode\n" if(exists
$Rbarcode{$temp[1]} and $Rbarcode{$temp[0]} ne $temp[3]);
	$sample{$temp[1]}{$temp[2]} = $temp[0];
	$length{$temp[1]} = length($temp[1]);
#	$linkPrimer{$temp[1]} = $temp[2];
	$Rbarcode{$temp[1]} = $temp[3];
	$reversePrimer{$temp[1]} = $temp[4];
}
close INC;


my $seqNum = 0;
open OUT,"> $outPrefix.split.fq" or die "$!\n";
open OUTE,"> $outPrefix.removed.fq" or die "$!\n";
if($inputFq =~ /gz$/){
	open INR,"gzip -dc $inputFq |" or die "$!\n";
}else{
	open INR,$inputFq or die "$!\n";
}
while(<INR>){
	$seqNum++;
	chomp;
	my $flag = 0;
	my $id = $_;
	$id =~ s/^@//;
	chomp(my $seq = <INR>);
	chomp(my $direction = <INR>);
	chomp(my $quality = <INR>);
	foreach my $Fbarcode(sort {$length{$a} <=> $length{$b}} keys %length){
		if($seq =~ /^$Fbarcode/i){
#			print "forward sequencing...\n";
			$flag = 1;
			my $reverse_seq = reverse $seq;
			$reverse_seq =~ tr/ATGC/TACG/;
			$reverse_seq =~ tr/atgc/tacg/;
			my $Rbarcode_mismatch = 0;
```

```
			for(my $i = 0;$i < length($Rbarcode{$Fbarcode});$i++){
				my $seq_base = substr($reverse_seq,$i,1);
				my $Rbarcode_base = substr($Rbarcode{$Fbarcode},$i,1);
#				$Rbarcode_mismatch++ unless($seq_base =~ /$Rbarcode_base/i);
				$Rbarcode_mismatch++ unless($abbrev{$Rbarcode_base} =~
/$seq_base/i);
			}
			if($Rbarcode_mismatch > 0){
				print OUTE "\@$id\tR-barcode\n$seq\n$direction\n$quality\n";
			}else{
				my $reFlag = 0;
				foreach my $linkPrimer(sort keys %{$sample{$Fbarcode}}){
					my $primer_mismatch = 0;
					for(my $i = 0;$i < length($linkPrimer);$i++){
						my $seq_base2 = substr($seq,length($Fbarcode) + $i,1);
						my $linkPrimer_base = substr($linkPrimer,$i,1);
						$primer_mismatch++ unless($abbrev{$linkPrimer_base} =~
/$seq_base2/i);
					}
					if($primer_mismatch <= $linkPrimer_th){
						my $temp_seq = substr($seq,length($Fbarcode) +
length($linkPrimer),length($seq) - length($Fbarcode) - length($linkPrimer) -
length($Rbarcode{$Fbarcode}) - length($reversePrimer{$Fbarcode}));
						my $temp_quality = substr($quality,length($Fbarcode) +
length($linkPrimer),length($quality) - length($Fbarcode) - length($linkPrimer) -
length($Rbarcode{$Fbarcode}) - length($reversePrimer{$Fbarcode}));
						print OUT
"\@$sample{$Fbarcode}{$linkPrimer}_$seqNum\t$id\torig_bc=$Fbarcode\tnew_bc=$F
barcode\tbc_diffs=0\n$temp_seq\n$direction\n$temp_quality\n";
						$reFlag = 1;
						last;
					}
				}
				print OUTE "\@$id\tprimer\n$seq\n$direction\n$quality\n"
unless($reFlag);
			}
			last;
		}
	}
	print OUTE "\@$id\tF-barcode\n$seq\n$direction\n$quality\n" unless($flag);
}
close INR;
close OUTE;
close OUT;
```

```
sub usage{
print <<EOD

	Description: split sequences for each sample by barcode
	Version: V1.20140214
	Contact: hua.chen\@majorbio.com

	usage: perl $0 -i merge.fq -f sample.config -l linkPrimer.cutoff -o out.prefix
		-i	merged fastq file,required
		-f	sample config file,include sampleID,barcode,link primer. required
		-l	link primer cutoff,default 2
		-o	output prefix,required
EOD
}
```

Table S1. Sample information.

Sample	Longitude (°)	Latitude (°)	Depth (m)	Environment	Ocean
DP003	110.20	17.00	1,250	Cold seep	Pacific Ocean
DP004	110.30	16.80	1,340	Cold seep	Pacific Ocean
DP005	110.30	17.20	1,100	Cold seep	Pacific Ocean
DP006	110.50	16.70	1,430	Cold seep	Pacific Ocean
DP007	111.00	17.00	1,450	Cold seep	Pacific Ocean
DP008	110.60	16.90	1,350	Cold seep	Pacific Ocean
DP009	111.10	16.80	1,500	Cold seep	Pacific Ocean
DP010	110.80	17.10	1,400	Cold seep	Pacific Ocean
DP016	137.52	8.06	4,159	Hadal trench	Pacific Ocean
DP017	137.55	8.06	4,993	Hadal trench	Pacific Ocean
DP018	137.60	8.10	6,173	Hadal trench	Pacific Ocean
DP019	137.60	8.00	6,582	Hadal trench	Pacific Ocean
DP020	137.80	8.00	6,682	Hadal trench	Pacific Ocean
DP021	138.68	9.66	5,100	Hadal trench	Pacific Ocean
DP022	138.82	9.65	4,500	Hadal trench	Pacific Ocean
DP025	63.76	3.66	3,400	Hydrothermal vent	Indian Ocean
DP026	63.72	3.69	3,550	Hydrothermal vent	Indian Ocean
DP027	-44.83	26.14	3,667	Hydrothermal vent	Atlantic Ocean
DP028	-44.99	14.75	3,048	Hydrothermal vent	Atlantic Ocean
DP029	-44.98	14.75	2,990	Hydrothermal vent	Atlantic Ocean
DP030	-14.53	-13.59	3,203	Hydrothermal vent	Atlantic Ocean
DP031	-14.31	-13.36	3,142	Hydrothermal vent	Atlantic Ocean
DP032	-14.52	-13.59	3,125	Hydrothermal vent	Atlantic Ocean
DP039	0.90	-3.10	3,534	Hydrothermal vent	Atlantic Ocean
DP040	-11.92	-19.35	2,597	Hydrothermal vent	Atlantic Ocean
DP041	-12.00	-19.32	2,607	Hydrothermal vent	Atlantic Ocean
DP042	-12.00	-19.27	2,761	Hydrothermal vent	Atlantic Ocean
DP046	-12.85	-18.04	3,307	Hydrothermal vent	Atlantic Ocean
DP047	-12.70	-18.49	2,146	Hydrothermal vent	Atlantic Ocean
DP048	-13.35	-15.17	2,782	Hydrothermal vent	Atlantic Ocean
DP049	-12.78	-18.49	2,146	Hydrothermal vent	Atlantic Ocean
DP050	49.70	-37.80	2,847	Hydrothermal vent	Indian Ocean
DP051	49.61	-37.90	1,933	Hydrothermal vent	Indian Ocean
DP052	49.83	-37.79	2,779	Hydrothermal vent	Indian Ocean
DP053	49.75	-37.79	2,295	Hydrothermal vent	Indian Ocean
DP054	48.20	-37.50	2,315	Hydrothermal vent	Indian Ocean
DP055	47.20	-38.20	2,284	Hydrothermal vent	Indian Ocean
DP057	46.76	-38.71	2,143	Hydrothermal vent	Indian Ocean
DP058	47.01	-38.68	2,826	Hydrothermal vent	Indian Ocean
DP059	-154.25	8.50	5,335	Ocean basin	Pacific Ocean
DP060	-153.33	10.54	5,133	Ocean basin	Pacific Ocean
DP061	-153.23	12.97	5,591	Ocean basin	Pacific Ocean
DP062	-154.82	9.75	5,302	Ocean basin	Pacific Ocean
DP063	-154.70	9.75	5,270	Ocean basin	Pacific Ocean
DP064	-154.44	9.75	5,267	Ocean basin	Pacific Ocean
DP065	-154.32	9.75	5,226	Ocean basin	Pacific Ocean
DP066	-154.25	9.68	5,246	Ocean basin	Pacific Ocean
DP067	-154.81	9.63	5,325	Ocean basin	Pacific Ocean

DP068	-154.57	9.63	5,130	Ocean basin	Pacific Ocean
DP069	-154.25	9.57	5,192	Ocean basin	Pacific Ocean
DP070	-154.38	9.56	5,253	Ocean basin	Pacific Ocean
DP071	-154.63	9.57	5,197	Ocean basin	Pacific Ocean
DP072	-154.69	9.50	5,233	Ocean basin	Pacific Ocean
DP073	-154.57	9.50	5,188	Ocean basin	Pacific Ocean
DP074	-154.25	9.50	5,240	Ocean basin	Pacific Ocean
DP075	-153.62	17.17	5,212	Ocean basin	Pacific Ocean
DP076	-152.39	17.83	5,140	Ocean basin	Pacific Ocean
DP077	-151.55	18.24	5,786	Ocean basin	Pacific Ocean
DP078	-174.50	9.72	5,868	Ocean basin	Pacific Ocean
DP081	49.10	-37.85	2,058	Hydrothermal vent	Indian Ocean
DP082	49.66	-37.79	2,625	Hydrothermal vent	Indian Ocean
DP083	-14.52	-13.60	2,949	Hydrothermal vent	Atlantic Ocean
DP085	63.94	-27.85	2,975	Hydrothermal vent	Indian Ocean
DP086	70.04	-25.32	2,434	Hydrothermal vent	Indian Ocean
DP087	54.37	-34.80	3,272	Hydrothermal vent	Indian Ocean
DP088	54.21	-34.97	3,206	Hydrothermal vent	Indian Ocean
DP089	54.59	-34.84	3,239	Hydrothermal vent	Indian Ocean
DP090	54.62	-34.84	2,335	Hydrothermal vent	Indian Ocean
DP091	50.37	-37.05	1,902	Hydrothermal vent	Indian Ocean
DP092	50.30	-37.70	2,028	Hydrothermal vent	Indian Ocean
DP093	50.20	-37.80	2,050	Hydrothermal vent	Indian Ocean
DP095	49.79	-37.79	2,987	Hydrothermal vent	Indian Ocean
DP096	52.99	-35.94	2,621	Hydrothermal vent	Indian Ocean
DP097	52.99	-35.99	2,645	Hydrothermal vent	Indian Ocean
DP098	49.55	-37.68	2,294	Hydrothermal vent	Indian Ocean
DP099	49.58	-37.73	2,828	Hydrothermal vent	Indian Ocean
DP100	47.42	-38.77	2,699	Hydrothermal vent	Indian Ocean
DP101	46.10	-37.60	4,371	Mid-ocean ridge	Indian Ocean
DP102	46.10	-37.70	5,166	Mid-ocean ridge	Indian Ocean
DP104	46.30	-37.70	5,679	Mid-ocean ridge	Indian Ocean
DP105	46.30	-37.80	5,665	Mid-ocean ridge	Indian Ocean
DP107	46.40	-37.70	5,955	Mid-ocean ridge	Indian Ocean
DP114	45.75	-37.26	2,298	Hydrothermal vent	Indian Ocean
DP117	52.67	-35.23	3,210	Hydrothermal vent	Indian Ocean
DP118	49.54	-37.71	2,502	Hydrothermal vent	Indian Ocean
DP119	49.45	-37.77	3,189	Hydrothermal vent	Indian Ocean
DP120	48.37	-38.28	2,864	Hydrothermal vent	Indian Ocean
DP121	48.50	-38.22	1,314	Hydrothermal vent	Indian Ocean
DP122	49.39	-37.71	2,380	Hydrothermal vent	Indian Ocean
DP123	48.44	-38.04	2,318	Hydrothermal vent	Indian Ocean
DP124	48.60	-38.10	2,774	Hydrothermal vent	Indian Ocean
DP125	48.29	-38.06	2,871	Hydrothermal vent	Indian Ocean
DP126	48.33	-38.15	2,519	Hydrothermal vent	Indian Ocean
DP127	46.58	-39.00	1,332	Hydrothermal vent	Indian Ocean
DP128	46.63	-38.87	3,251	Hydrothermal vent	Indian Ocean
DP129	55.36	-34.44	3,057	Hydrothermal vent	Indian Ocean
DP130	55.21	-34.46	2,556	Hydrothermal vent	Indian Ocean
DP131	55.16	-34.44	3,332	Hydrothermal vent	Indian Ocean
DP132	49.81	-37.94	1,535	Hydrothermal vent	Indian Ocean
DP134	49.90	-37.80	1,496	Hydrothermal vent	Indian Ocean

DP135	49.90	-37.90	1,486	Hydrothermal vent	Indian Ocean
DP136	49.80	-37.80	1,512	Hydrothermal vent	Indian Ocean
DP137	49.70	-37.90	1,513	Hydrothermal vent	Indian Ocean
DP138	49.60	-37.50	1,511	Hydrothermal vent	Indian Ocean
DP139	127.20	27.50	1,275	Hadal trench	Pacific ocean
DP140	127.30	27.70	1,308	Hadal trench	Pacific ocean

Table S2. 16S rRNA gene sequencing data of bacteria and archaea.

Sample	Bacteria			Archaea		
	Reads	OTU	coverage	Reads	OTU	coverage
DP003	38881	2364	0.988143	24070	270	0.996801
DP004	53737	2347	0.994045	23562	292	0.996308
DP005	32826	377	0.992597	18264	326	0.994908
DP006	54450	1325	0.988944	24219	255	0.996779
DP007	47137	1073	0.996839	7433	140	0.993139
DP008	32301	549	0.99127	20258	189	0.997137
DP009	31094	1253	0.983534	19992	298	0.996098
DP010	32128	645	0.989791	23392	193	0.997606
DP016	54041	368	0.999408	682	48	0.972141
DP017	49223	231	0.999614	3775	53	0.994702
DP018	46151	463	0.996771	15260	157	0.996592
DP019	51744	508	0.997623	13967	159	0.996205
DP020	48695	1050	0.996386	33368	176	0.998292
DP021	36076	431	0.996701	8718	67	0.998394
DP022	57098	354	0.999107	20933	166	0.998185
DP025	37602	1245	0.995612	5233	134	0.991592
DP026	54289	1664	0.996832	3870	104	0.991473
DP027	52692	1683	0.994933	8655	130	0.995494
DP028	29044	1734	0.986469	5732	133	0.991277
DP029	35083	2177	0.983582	18678	218	0.99652
DP030	33388	1086	0.996496	647	23	0.987635
DP031	49129	1461	0.997191	7684	148	0.993363
DP032	48442	1861	0.995562	27943	104	0.998819
DP039	54555	1543	0.996554	58	24	0.758621
DP040	53822	1372	0.997845	1186	115	0.96543
DP041	47581	826	0.99813	9190	115	0.995756
DP042	36247	385	0.998731	26824	171	0.998285
DP046	52700	1472	0.997932	851	23	0.985899
DP047	44542	1860	0.989785	36037	305	0.997558
DP048	38379	1126	0.994294	1773	73	0.988156
DP049	53609	1263	0.996661	1198	22	0.992487
DP050	52565	822	0.996956	1571	67	0.989815
DP051	45462	853	0.997184	5332	116	0.993811
DP052	49085	237	0.997657	4370	26	0.997254
DP053	30040	909	0.995872	10803	121	0.995742
DP054	34451	3171	0.970857	38978	333	0.997229
DP055	48660	1901	0.994143	24668	229	0.997892
DP057	40507	1047	0.99136	32040	178	0.998658
DP058	41353	1369	0.990158	9678	75	0.9969
DP059	53728	2610	0.987828	11715	130	0.996159
DP060	38467	762	0.997686	329	33	0.966565
DP061	48005	326	0.999229	31661	234	0.998042
DP062	44644	502	0.995095	22363	201	0.997719
DP063	36569	744	0.997812	20254	206	0.997038
DP064	52671	780	0.995937	16588	115	0.99783
DP065	55097	863	0.995408	31641	168	0.998641
DP066	51983	402	0.998538	36793	230	0.998369
DP067	47394	1462	0.992003	23453	244	0.997399
DP068	65931	991	0.994858	13857	168	0.996464

DP069	34589	1009	0.989245	208	25	0.932692
DP070	38948	2100	0.992041	25721	202	0.99825
DP071	44793	1917	0.993593	357	42	0.957983
DP072	29182	1844	0.983517	41151	319	0.998129
DP073	49174	822	0.997234	22003	191	0.997091
DP074	37127	602	0.998034	44304	289	0.998307
DP075	42641	442	0.994395	16882	206	0.996683
DP076	30620	1406	0.993926	37763	241	0.997934
DP077	52065	307	0.99952	1961	36	0.990311
DP078	43613	824	0.991723	49119	269	0.998738
DP081	48745	762	0.992512	34725	208	0.998445
DP082	39110	607	0.995167	504	19	0.990079
DP083	40247	839	0.996372	4794	83	0.994159
DP085	41162	1459	0.996502	12598	194	0.995952
DP086	41120	877	0.996328	1580	66	0.987342
DP087	35119	189	0.997779	27280	588	0.993988
DP088	37633	743	0.996891	9050	164	0.994696
DP089	50281	866	0.992701	1413	11	0.997169
DP090	46469	888	0.99703	4159	122	0.989421
DP091	36575	403	0.996172	1239	87	0.982244
DP092	44609	1005	0.992266	5786	37	0.99758
DP093	51239	383	0.999278	1053	58	0.988604
DP095	56858	709	0.996166	2995	38	0.994658
DP096	95434	1496	0.99847	15907	204	0.997171
DP097	52646	269	0.998803	11797	257	0.995338
DP098	43411	1047	0.997144	252	43	0.960317
DP099	58522	1231	0.99566	4856	60	0.998147
DP100	33008	383	0.992881	1297	77	0.983809
DP101	40226	1040	0.988689	18992	200	0.997157
DP102	45263	927	0.994057	32285	194	0.998111
DP104	54369	987	0.998087	14743	100	0.997626
DP105	40804	733	0.991104	33325	256	0.997839
DP107	54038	806	0.993597	8615	82	0.996982
DP114	30896	1022	0.98482	18352	144	0.997439
DP117	36472	807	0.998382	19146	192	0.997336
DP118	30995	1100	0.994515	264	15	0.973485
DP119	55105	471	0.995881	16768	88	0.997793
DP120	51847	1149	0.998129	817	37	0.98164
DP121	54120	1489	0.995935	8570	154	0.994982
DP122	55867	2115	0.994147	43919	294	0.998634
DP123	45504	807	0.990836	13356	143	0.996181
DP124	46262	814	0.998076	18530	113	0.998111
DP125	29847	871	0.994773	10625	123	0.997271
DP126	45760	1072	0.991455	19752	269	0.996203
DP127	37431	1498	0.993374	10322	228	0.9938
DP128	29648	829	0.989375	17287	98	0.99838
DP129	46572	701	0.99242	5502	130	0.990549
DP130	37133	375	0.995045	16996	138	0.996999
DP131	32201	378	0.998634	6203	80	0.995325
DP132	45949	1188	0.991469	1483	49	0.98584
DP134	39495	1238	0.986226	23995	188	0.997333
DP135	56166	1330	0.998718	56	12	0.910714

DP136	51998	1936	0.99223	11473	202	0.994857
DP137	29108	1504	0.982926	24313	306	0.99671
DP138	38072	564	0.997899	4034	160	0.988349
DP139	72670	318	0.998927	7654	27	0.999477
DP140	54446	728	0.995996	25312	132	0.998933

Table S3. Classified bacterial genera of 106 deep-sea sediment samples.

Classified bacterial genera
Bacteroides, Pir4 lineage, Lactobacillus, Planctomyces, Parabacteroides, Nitrosomonas, Coxiella, Faecalibacterium, [Ruminococcus] torques group, Barnesiella, Urania-1B-19 marine sediment group, Rhodopirellula, Blastopirellula, H16, Nitrospira, Pannonibacter, Unclassified, Granulosicoccus, Nocardioides, Erysipelatoclostridium, Aquibacter, [Eubacterium] coprostanoligenes group, Pelagibius, Nitrospina, Ruminiclostridium 9, Pseudomonas, Ruminococcaceae UCG-014, Alistipes, Filomicrobium, Lachnospiraceae NK4A136 group, Alloprevotella, Megamonas, Bythopirellula, Escherichia-Shigella, Butyricicoccus, Ochrobactrum, Shuttleworthia, Blautia, Rubripirellula, Anaerofilum, Enorma, Enterococcus, Anaerotruncus, Subdoligranulum, Ruminiclostridium 5, Caldithrix, Haliangium, Reichenbachiella, Schleiferia, Sutterella, OM60(NOR5) clade, Aquicella, Cohaesibacter, Phascolarctobacterium, Megasphaera, Ruminococcaceae UCG-013, Bacillus, Odoribacter, Eisenbergiella, Lachnoclostridium, Anderseniella, Ruminiclostridium, Synechococcus, Collinsella, Delftia, Magnetospira, Ulvibacter, Candidatus Microthrix, Lutibacter, Streptococcus, Prevotellaceae UCG-001, Peredibacter, Vibrio, Oscillibacter, PAUC26f, Ruminococcaceae UCG-005, Truepera, Arthrobacter, Chroococcidiopsis, Enterorhabdus, Pseudohongiella, Rhizobium, Ruminococcaceae UCG-010, Turicibacter, Brevundimonas, Haliea, Marinobacterium, Pirellula, [Eubacterium] xylanophilum group, Faecalibaculum, Lentisphaera, RB41, Ruminococcaceae UCG-009, Acinetobacter, Ensifer,

Oceanirhabdus, *Planomicrobium*, *Portibacter*, *Rhodobium*, *SM1A02*, *Sva0081 sediment group*, *Winogradskyella*, *Ichthyenterobacterium*, *Labrenzia*, *Legionella*, *Pedobacter*, *Phenylobacterium*, *Solirubrobacter*, *Achromobacter*, *Actinomyces*, *BD1-7 clade*, *Butyricimonas*, *Cerasicoccus*, *Colwellia*, *Desulfatiglans*, *Dichotomicrobium*, *Enterobacter*, *Halobacteriovorax*, *Roseobacter clade NAC11-7 lineage*, *Sulfitobacter*, *Yersinia*, *[Eubacterium] hallii group*, *Candidatus Tenderia*, *Coprococcus 1*, *Flavonifractor*, *Lachnospiraceae UCG-001*, *Leuconostoc*, *Marinobacter*, *Massilia*, *Olleya*, *Paraprevotella*, *Roseomonas*, *Sedimentitalea*, *Acanthopleuribacter*, *Bosea*, *Dietzia*, *Ectothiorhodospira*, *Fluviicola*, *G55*, *Hyphomicrobium*, *Jeotgalibacillus*, *Lutimonas*, *OM27 clade*, *Oscillospira*, *Parasphingopyxis*, *Pediococcus*, *Phaselicystis*, *Slackia*, *Sphingorhabdus*, *Succinatimonas*, *Tepidibacter*, *Thiobacillus*, *Thiohalophilus*, *Turneriella*, *marine group*, *Amaricoccus*, *Bergeyella*, *Blastocatella*, *Bradyrhizobium*, *Brevibacterium*, *Candidatus Saccharimonas*, *Crocinitomix*, *Ekhidna*, *Filimonas*, *Fulvivirga*, *MSBL7*, *Oerskovia*, *Opitutus*, *Paenibacillus*, *Phyllobacterium*, *Propionibacterium*, *Shewanella*, *Stenotrophomonas*, *Thermincola*, *Thioalkalispira*, *Anaerostipes*, *Burkholderia-Paraburkholderia*, *CL500-3*, *Calothrix*, *Candidatus Omnitrophus*, *Christensenellaceae R-7 group*, *Clostridium sensu stricto*, *Desulfuromonas*, *Dongia*, *Emcibacter*, *Erythrobacter*, *Exiguobacterium*, *FS140-16B-02 marine group*, *Flavirhabdus*, *Gaiella*, *Gemmobacter*, *Halioglobus*, *Jannaschia*, *Kangiella*, *Kocuria*, *Lactococcus*, *Moritella*, *Mycobacterium*, *Oceanibaculum*, *Paracoccus*, *Pelagicoccus*, *Phormidium*, *Pseudofulvibacter*, *Roseburia*, *SEEP-SRB1*, *Salimesophilobacter*,

Sneathiella, Sphingobacterium, Sphingomonas, Spirochaeta 2, Staphylococcus, Thalassobacillus, Thiohalorhabdus, Vallitalea, 11-24, 12up, AEGEAN-169 marine group, AKIW659, AKYG587, Abiotrophia, Acaryochloris, Acetatifactor, Acetitomaculum, Acetoanaerobium, Acetobacter, Acetobacterium, Acetobacteroides, Acholeplasma, Acidaminobacter, Acidaminococcus, Acidibacter, Acidicaldus, Acidicapsa, Acidiphilium, Acidisoma, Acidisphaera, Acidithiobacillus, Acidobacterium, Acidocella, Acidothermus, Acidovorax, Actinoallomurus, Actinobacillus, Actinocatenispora, Actinomadura, Actinomycetospora, Actinophytocola, Actinoplanes, Actinospica, Adlercreutzia, Advenella, Aequorivita, Aerococcus, Aeromicrobium, Aeromonas, Aestuariicella, Afipia, Agromyces, Akkermansia, Alcaligenes, Alcanivorax, Algibacter, Algiphilus, Algoriphagus, Alicycliphilus, Aliidiomarina, Aliihoeflea, Alishewanella, Alkalibacillus, Alkalibacter, Alkaliphilus, Alkanibacter, Allobaculum, Alpinimonas, Altererythrobacter, Alterococcus, Alteromonas, Amantichitinum, Aminiphilus, Aminobacter, Aminobacterium, Aminomonas, Ammoniphilus, Amphritea, Amycolatopsis, Anabaenopsis, Anaerobiospirillum, Anaerobranca, Anaerocella, Anaerococcus, Anaerofustis, Anaerolinea, Anaerolineaceae UCG-001, Anaeromyxobacter, Anaeroplasma, Anaerosinus, Anaerosporobacter, Anaerovibrio, Anaerovorax, Ancylobacter, Anoxybacillus, Apibacter, Aquaspirillum, Aquimarina, Aquimonas, Arcobacter, Arenibacter, Arenicella, Arenimonas, Aridibacter, Armatimonas, Arsenophonus, Asaia, Asticcacaulis, Atopobium, Atopostipes, Aurantimonas, Aureimonas, Aureispira, Azoarcus, Azospira, Azovibrio, Bacteriovorax, Balneola,

Bartonella, Bauldia, Bdellovibrio, Beggiatoa, Bellilinea, Belnapia, Bifidobacterium, Bilophila, Blastococcus, Blastomonas, Blattabacterium, Blvii28 wastewater-sludge group, Bordetella, Brachybacterium, Brachymonas, Brevibacillus, Breznakia, Brochothrix, Bryobacter, Buchnera, Butyrivibrio, Butyrivibrio 2, C1-B045, CL500-29 marine group, CPla-4 termite group, Caldicoprobacter, Caldimonas, Caldisericum, Campylobacter, Candidatus Accumulibacter, Candidatus Actinomarina, Candidatus Allobeggiatoa, Candidatus Alysiosphaera, Candidatus Amoebophilus, Candidatus Amphibiichlamydia, Candidatus Anammoximicrobium, Candidatus Aquiluna, Candidatus Aquirestis, Candidatus Arthromitus, Candidatus Bacilloplasma, Candidatus Brocadia, Candidatus Caldatribacterium, Candidatus Captivus, Candidatus Chloroploca, Candidatus Cloacamonas, Candidatus Competibacter, Candidatus Endoecteinascidia, Candidatus Endomicrobium, Candidatus Gigarickettsia, Candidatus Gortzia, Candidatus Hepatincola, Candidatus Hepatoplasma, Candidatus Koribacter, Candidatus Limnoluna, Candidatus Methylopumilus, Candidatus Nostocoida, Candidatus Odyssella, Candidatus Protochlamydia, Candidatus Riegeria, Candidatus Scalindua, Candidatus Soleaferrea, Candidatus Solibacter, Candidatus Stoquefichus, Candidatus Thiobios, Candidatus Trichorickettsia, Candidatus Xiphinematobacter, Capnocytophaga, Caproiciproducens, Carboxylicivirga, Castellaniella, Catabacter, Catenibacterium, Catenisphaera, Catenulispora, Catonella, Caulobacter, Cellulophaga, Cellulosilyticum, Cellvibrio, Cesiribacter, Cetobacterium, Chitinibacter, Chitinimonas, Chitinivorax, Chitinophaga, Christensenella, Chromobacterium,

Chryseobacterium, *Chryseolinea*, *Chthoniobacter*, *Chthonomonas*, *Citreicella*, *Cloacibacillus*, *Cloacibacterium*, *Clostridiisalibacter*, *Clostridium sensu stricto 1*, *Clostridium sensu stricto 11*, *Clostridium sensu stricto 12*, *Clostridium sensu stricto 13*, *Clostridium sensu stricto 15*, *Clostridium sensu stricto 3*, *Clostridium sensu stricto 5*, *Clostridium sensu stricto 6*, *Clostridium sensu stricto 9*, *Cobetia*, *Cocleimonas*, *Cohnella*, *Comamonas*, *Conexibacter*, *Constrictibacter*, *Coprobacter*, *Coprococcus 2*, *Coprococcus 3*, *Coraliomargarita*, *Coriobacteriaceae UCG-002*, *Corynebacterium*, *Corynebacterium 1*, *Craurococcus*, *Crenothrix*, *Crinalium*, *Croceicoccus*, *Cryomorpha*, *Cryptanaerobacter*, *Cupriavidus*, *Cyanothece*, *Cyclobacterium*, *Cycloclasticus*, *Cytophaga*, *DS-100*, *Dactylosporangium*, *Dasania*, *Dechloromonas*, *Deferrisoma*, *Defluviicoccus*, *Defluviimonas*, *Defluviitaleaceae UCG-011*, *Deinococcus*, *Demequina*, *Denitratisoma*, *Desulfatirhabdium*, *Desulfitibacter*, *Desulfobacca*, *Desulfobulbus*, *Desulfocapsa*, *Desulfocarbo*, *Desulfocella*, *Desulfococcus*, *Desulfofrigus*, *Desulfofustis*, *Desulfomicrobium*, *Desulfonema*, *Desulfopila*, *Desulforhabdus*, *Desulfosarcina*, *Desulfosporosinus*, *Desulfotomaculum*, *Desulfovibrio*, *Desulfurispora*, *Desulfurivibrio*, *Desulfurobacterium*, *Desulfuromusa*, *Dethiosulfatibacter*, *Dethiosulfovibrio*, *Devosia*, *Dialister*, *Diaminobutyricimonas*, *Diaphorobacter*, *Dielma*, *Dinghuibacter*, *Diplorickettsia*, *Diplosphaera*, *Dokdonella*, *Dolosigranulum*, *Dorea*, *Draconibacterium*, *Dyadobacter*, *Dyella*, *Dysgonomonas*, *Edaphobacter*, *Eggerthella*, *Ehrlichia*, *Elev-16S-1166*, *Elioraea*, *Elusimicrobium*, *Empedobacter*, *Emticicia*, *Endozoicomonas*, *Enhydrobacter*, *Epilithonimonas*, *Epulopiscium*, *Ercella*, *Erwinia*,

Erysipelothrix, Erysipelotrichaceae UCG-003, Erysipelotrichaceae UCG-004, Eubacterium, Eudoraea, Euzebya, Ezakiella, Faecalicoccus, Faecalitalea, Family XIII AD3011 group, Family XIII UCG-001, Family XIII UCG-002, Fastidiosipila, Ferritrophicum, Ferrovibrio, Ferruginibacter, Fibrella, Fibrobacter, Filifactor, Fimbriimonas, Finegoldia, Flaviflexus, Flavihumibacter, Flavitalea, Flavobacterium, Flectobacillus, Flexithrix, Fodinicola, Fontibacter, Fonticella, Fretibacterium, Friedmanniella, Frigoribacterium, Frondihabitans, Fusibacter, Fusicatenibacter, Fusobacterium, GOUTB8, Galbibacter, Gallibacterium, Gallicola, Gardnerella, Gelria, Gemella, Geminicoccus, Gemmata, Gemmatimonas, Gemmatirosa, Geobacillus, Geobacter, Geomicrobium, Geopsychrobacter, Georgenia, Geothrix, Gilliamella, Gillisia, Gilvibacter, Glycocaulis, Glycomyces, Gordonia, Gordonibacter, Gracilimonas, Gramella, Granulicella, Haematospirillum, Haemophilus, Haliscomenobacter, Halomonas, Halothiobacillus, Hamadaea, Helicobacter, Henriciella, Herbidospora, Herpetosiphon, Hippea, Hirschia, Holdemanella, Holdemania, Holophaga, Howardella, Hungatella, Hydrocarboniphaga, Hydrogenimonas, Hydrogenispora, Hydrogenoanaerobacterium, Hydrogenophilus, Hymenobacter, Hyphomonas, I-8, Iamia, Ideonella, Idiomarina, Ignavibacterium, Ignavigranum, Inhella, Inquilinus, Intestinibacter, Isosphaera, Jatrophihabitans, Jejudonia, Jeotgalicoccus, Johnsonella, Jonquetella, Joostella, Kaistia, Kandleria, Kineosporia, Kitasatospora, Kordiimonas, Koukoulia, Kribbella, Kriegella, Ktedonobacter, Kurthia, Kushneria, Kutzneria, Labrys, Lachnoanaerobaculum, Lachnoclostridium 10, Lachnoclostridium 5, Lachnospira,

Lachnospiraceae AC2044 group, Lachnospiraceae FCS020 group, Lachnospiraceae FE2018 group, Lachnospiraceae NC2004 group, Lachnospiraceae ND3007 group, Lachnospiraceae NK3A20 group, Lachnospiraceae NK4B4 group, Lachnospiraceae UCG-004, Lachnospiraceae UCG-006, Lachnospiraceae UCG-008, Lachnospiraceae UCG-010, Lachnospiraceae XPB1014 group, Lacibacter, Lapillicoccus, Larkinella, Lautropia, Lawsonella, Leadbetterella, Leeia, Leeuwenhoekiella, Leifsonia, Lentimonas, Leptolinea, Leptolyngbya, Leptonema, Leptospira, Leptospirillum, Leptotrichia, Leucobacter, Leucothrix, Levilinea, Lewinella, Limnobacter, Limnothrix, Lishizhenia, Litoribacillus, Litoricola, Litorilinea, Litorimonas, Loktanella, Longilinea, Longispora, Luedemannella, Luteimonas, Luteolibacter, Lutispora, Lyngbya, Lysobacter, MWH-UniP1 aquatic group, Macellibacteroides, Macrococcus, Maribacter, Maricaulis, Marine Methylotrophic Group 3, Marinicella, Marinifilum, Marinilabilia, Marinilactibacillus, Marinimicrobium, Mariniphaga, Mariniradius, Marinococcus, Marinomonas, Marinoscillum, Mariprofundus, Marispirillum, Maritimimonas, Marivirga, Marixanthomonas, Marmoricola, Marvinbryantia, Mastigocladopsis, Meganema, Meiothermus, Melioribacter, Melittangium, Merismopedia, Mesoaciditoga, Mesoflavibacter, Mesonia, Mesorhizobium, Mesotoga, Methylobacillus, Methylobacterium, Methylocaldum, Methylocella, Methyloparacoccus, Methylophaga, Methylorosula, Methylotenera, Methyloversatilis, Microbacter, Microbacterium, Micrococcus, Microcoleus, Microlunatus, Micromonospora, Microvirga, Mitsuokella, Mizugakiibacter, Mobilitalea, Mobiluncus, Mogibacterium, Moheibacter, Moraxella, Motiliproteus,

Mucilaginibacter, Mucinivorans, Mucispirillum, Muricauda, Muriicola, Mycoavidus, Mycoplasma, Myroides, NS10 marine group, NS3a marine group, NS4 marine group, NS5 marine group, Nafulsella, Nakamurella, Nannocystis, Natranaerovirga, Naumannella, Negadavirga, Neisseria, Neochlamydia, Neorhizobium, Neorickettsia, Nesterenkonia, Nevskia, Niastella, Nibrella, Nitratireductor, Nitriliruptor, Nitrolancea, Nitrospirillum, Nocardia, Nonlabens, Nonomuraea, Nordella, Nostoc, Novispirillum, Novosphingobium, Nubsella, OM75 clade, Oceanimonas, Oceanobacillus, Oceanobacter, Oceanococcus, Oceanospirillum, Ohtaekwangia, Oligella, Oligoflexus, Olsenella, Oribacterium, Ornatilinea, Oscillochloris, Owenweeksia, PS-B30, Paenarthrobacter, Paeniglutamicibacter, Paenisporosarcina, Paludibacter, Paludibacterium, Paludibaculum, Panacagrimonas, Papillibacter, Paraclostridium, Parafilimonas, Paramoritella, Parapedobacter, Parapusillimonas, Parasutterella, Parvibacter, Parvibaculum, Parvimonas, Pasteurella, Patulibacter, Paucibacter, Pedomicrobium, Pelagibacterium, Pelolinea, Pelomonas, Pelosinus, Pelotomaculum, Peptoclostridium, Peptococcus, Peptoniphilus, Peptostreptococcus, Perlucidibaca, Persicitalea, Petrimonas, Phaeodactylibacter, Phocaeicola, Photobacterium, Phreatobacter, Phycisphaera, Pibocella, Pir2 lineage, Piscicoccus, Planktosalinus, Planktothrix, Planosporangium, Plantactinospora, Pleomorphomonas, Plesiocystis, Plesiomonas, Pleurocapsa, Polaribacter 2, Polaromonas, Polycyclovorans, Polymorphobacter, Polynucleobacter, Pontibacter, Porphyromonas, Porticoccus, Prevotella, Prevotella 1, Prevotella 2, Prevotella 6, Prevotella 7, Prevotella 9, Prevotellaceae Ga6A1 group, Prevotellaceae NK3B31

group, Prevotellaceae UCG-003, Prevotellaceae UCG-004, Procabacter, Prochlorococcus, Prochlorothrix, Profundimonas, Prolixibacter, Promicromonospora, Propionicicella, Propioniciclava, Prosthecomicrobium, Proteiniphilum, Proteocatella, Proteus, Pseudahrensia, Pseudarcicella, Pseudenhygromyxa, Pseudoalteromonas, Pseudobutyrivibrio, Pseudochelatococcus, Pseudofulvimonas, Pseudohaliea, Pseudokineococcus, Pseudolabrys, Pseudonocardia, Pseudopedobacter, Pseudoroseovarius, Pseudoxanthomonas, Psychrilyobacter, Psychrobacter, Psychrobium, Psychromonas, Pusillimonas, Quadrisphaera, Quinella, Ralstonia, Ramlibacter, Rapidithrix, Rathayibacter, Reyranella, Rheinheimera, Rhizocola, Rhizomicrobium, Rhizorhapis, Rhodanobacter, Rhodobacter, Rhodoblastus, Rhodocista, Rhodococcus, Rhodocytophaga, Rhodomicrobium, Rhodoplanes, Rhodovarius, Rhodovastum, Rickettsiella, Rikenella, Rikenellaceae RC9 gut group, Robiginitalea, Romboutsia, Roseiarcus, Roseiflexus, Roseivirga, Roseivivax, Roseococcus, Roseovarius, Rs-D38 termite group, Rubellimicrobium, Rubribacterium, Rubricoccus, Rubrobacter, Ruminiclostridium 1, Ruminiclostridium 6, Ruminobacter, Ruminococcaceae NK4A214 group, Ruminococcaceae UCG-002, Ruminococcaceae UCG-004, Ruminococcaceae UCG-007, Ruminococcaceae UCG-008, Ruminococcaceae UCG-011, Ruminococcaceae V9D2013 group, Ruminococcus 1, Ruminococcus 2, Ruminofilibacter, Rummeliibacillus, Runella, SEEP-SRB4, Saccharomonospora, Saccharopolyspora, Saccharothrix, Salegentibacter, Salinicola, Salinimicrobium, Salinisphaera, Sandaracinobacter, Sandaracinus, Sarcina, Schlesneria,

Sedimentibacter, *Sediminibacterium*, *Segetibacter*, *Selenomonas*, *Selenomonas 3*, *Sellimonas*, *Senegalimassilia*, *Seohaeicola*, *Serinicoccus*, *Serratia*, *Shinella*, *Silanimonas*, *Simiduia*, *Singulisphaera*, *Skermanella*, *Smaragdicoccus*, *Smithella*, *Solitalea*, *Solobacterium*, *Sorangium*, *Sphaerochaeta*, *Sphingobium*, *Sphingopyxis*, *Sphingosinicella*, *Spiroplasma*, *Spirosoma*, *Spirulina*, *Spongiimonas*, *Sporichthya*, *Sporocytophaga*, *Stanieria*, *Stella*, *Stenotrophobacter*, *Steroidobacter*, *Sterolibacterium*, *Stomatobaculum*, *Streptacidiphilus*, *Streptobacillus*, *Streptomyces*, *Succiniclasticum*, *Succinivibrio*, *Sulfobacillus*, *Sulfurifustis*, *Sulfurimonas*, *Sulfuritalea*, *Sulfurospirillum*, *Sulfurovum*, *Sunxiuqinia*, *Symbiobacterium*, *Syntrophobacter*, *Syntrophococcus*, *Syntrophomonas*, *Syntrophorhabdus*, *Tagaea*, *Tahibacter*, *Taibaiella*, *Tamlana*, *Tannerella*, *Telmatocola*, *Telmatospirillum*, *Temperatibacter*, *Tenacibaculum*, *Tepidiphilus*, *Terasakiella*, *Termite planctomycete cluster*, *Terrimonas*, *Terrisporobacter*, *Tessaracoccus*, *Tetrasphaera*, *Thalassospira*, *Thalassotalea*, *Thauera*, *Thermacetogenium*, *Thermaerobacter*, *Thermoanaerobaculum*, *Thermobacillus*, *Thermobifida*, *Thermoflexus*, *Thermomonas*, *Thermomonospora*, *Thermopolyspora*, *Thermovenabulum*, *Thermovirga*, *Thermovum*, *Thermus*, *Thioalbus*, *Thiogranum*, *Thiomicrospira*, *Thioreductor*, *Thiothrix*, *Tissierella*, *Tistlia*, *Tolumonas*, *Treponema*, *Treponema 2*, *Trichococcus*, *Tropicimonas*, *Trueperella*, *Tumebacillus*, *Tyzzerella*, *Tyzzerella 3*, *Uliginosibacterium*, *Undibacterium*, *Vagococcus*, *Variibacter*, *Variovorax*, *Veillonella*, *Vicinamibacter*, *Victivallis*, *Vitellibacter*, *Vogesella*, *Vulcaniibacterium*, *Vulgatibacter*, *Waddlia*, *Weissella*, *Wolbachia*, *Woodsholea*, *Xanthobacter*, *Xenophilus*,

Yonghaparkia, Youhaiella, ZD0417 marine group, Zavarzinella, Zoogloea, Zunongwangia, Zymomonas, [Acetivibrio] ethanolgignens group, [Agitococcus] lubricus group, [Anaerorhabdus] furcosa group, [Bacteroides] pectinophilus group, [Clostridium] innocuum group, [Desulfobacterium] catecholicum group, [Eubacterium] brachy group, [Eubacterium] fissicatena group, [Eubacterium] nodatum group, [Eubacterium] rectale group, [Eubacterium] ruminantium group, [Eubacterium] saphenum group, [Eubacterium] ventriosum group, [Ruminococcus] gauvreauii group, dgA-11 gut group, endosymbionts, hgcI clade, hoa5-07d05 gut group, p-1088-a5 gut group, possible genus 04, vadinBC27 wastewater-sludge group

Table S4. Classified archaeal genera of 106 deep-sea sediment samples.

Classified archaeal genera
Candidatus Nitrosopumilus, Methanopyrus, Candidatus Nitrososphaera, Candidatus Nitrosopelagicus, Methanobrevibacter, Methanocorpusculum, Methanosaeta, Thermococcus, Candidatus Nitrosotalea, Methanosarcina, Halococcus, Haladaptatus, Methanosphaera, Methanobacterium, Cenarchaeum, Halomicrobium, Methanothermococcus, Candidatus Aciduliprofundum, Thermofilum, Methanothermobacter, Halostagnicola, Rice Cluster I, Candidatus Nitrosoarchaeum, Methanomassiliicoccus, Methanocella, Halorubrum, Halomarina, Halogeometricum, Candidatus Korarchaeum, Halalkalicoccus, Candidatus Methanoperedens, Natronomonas, Halorientalis, Haloterrigena, Halorussus, Halarchaeum, Haloarcula, Haloferax, Halovivax, Natronolimnobius
